# Characterisation of the *Paenarthrobacter nicotinovorans* ATCC 49919 genome and identification of several strains harbouring a highly syntenic *nic*-genes cluster

**DOI:** 10.1186/s12864-023-09644-3

**Published:** 2023-09-11

**Authors:** Amada El-Sabeh, Andreea-Mihaela Mlesnita, Iustin-Tiberius Munteanu, Iasmina Honceriu, Fakhri Kallabi, Razvan-Stefan Boiangiu, Marius Mihasan

**Affiliations:** 1 Faculty of Biology, Alexandru Ioan Cuza University of Iași, Iași, Romania; 2 Laboratory of Human Molecular Genetics, Faculty of Medicine of Sfax, University of Sfax, Sfax, Tunisia

**Keywords:** Paenarthrobacter, Nicotine metabolism, Evolution, Plasmids, Catabolic transposon

## Abstract

**Background:**

*Paenarthrobacter nicotinovorans* ATCC 49919 uses the pyridine-pathway to degrade nicotine and could provide a renewable source of precursors from nicotine-containing waste as well as a model for studying the molecular evolution of catabolic pathways and their spread by horizontal gene transfer via soil bacterial plasmids.

**Results:**

In the present study, the strain was sequenced using the Illumina NovaSeq 6000 and Oxford Nanopore Technology (ONT) MinION platforms. Following hybrid assembly with Unicycler, the complete genome sequence of the strain was obtained and used as reference for whole-genome-based phylogeny analyses. A total of 64 related genomes were analysed; five *Arthrobacter* strains showed both digital DNA-DNA hybridization and average nucleotide identity values over the species threshold when compared to *P. nicotinovorans* ATCC 49919. Five plasmids and two contigs belonging to *Arthrobacter* and *Paenarthrobacter* strains were shown to be virtually identical with the pAO1 plasmid of *Paenarthrobacter nicotinovorans* ATCC 49919. Moreover, a highly syntenic *nic*-genes cluster was identified on five plasmids, one contig and three chromosomes. The *nic*-genes cluster contains two major locally collinear blocks that appear to form a putative catabolic transposon. Although the origins of the *nic*-genes cluster and the putative transposon still elude us, we hypothesise here that the ATCC 49919 strain most probably evolved from *Paenarthrobacter* sp. YJN-D or a very closely related strain by acquiring the pAO1 megaplasmid and the nicotine degradation pathway.

**Conclusions:**

The data presented here offers another snapshot into the evolution of plasmids harboured by *Arthrobacter* and *Paenarthrobacter* species and their role in the spread of metabolic traits by horizontal gene transfer among related soil bacteria.

**Supplementary Information:**

The online version contains supplementary material available at 10.1186/s12864-023-09644-3.

## Background

*Paenarthrobacter nicotinovorans* ATCC 49919 is an aerobic Gram-positive soil bacterium that can use nicotine as a carbon source. On media containing this alkaloid, the strain produces a characteristic blue pigment known as nicotine blue (NB or 4,4′,5,5′-tetrahydroxy-3,3′-diazadiphenoquinone-(2,2′)). First referenced under the name *Arthrobacter oxidans* [1, 2], the strain was sequentially renamed *Arthrobacter nicotinovorans* [3] and finally *Paenarthrobacter nicotinovorans* [4]. The pyridine-pathway used by this bacterium to degrade nicotine is historically the first, hence the most thoroughly studied bacterial catabolic pathway for this alkaloid [5, 6]. All the intermediates, steps and enzymes involved in the conversion of nicotine to NB are well established and there is available data on the nicotine pathway regulation. The genes responsible for nicotine degradation are grouped in a *nic*-genes cluster located on pAO1, a 165 kb catabolic megaplasmid [7]. The cluster is organized into several gene modules [8] responsible for the catabolism of L- and D-nicotine to α-ketoglutarate, succinate, methylamine and NB. While NB and methylamine accumulate in the growth medium, we have shown that α-ketoglutarate and succinate are integrated into the Krebs cycle and support cell growth [9, 10].

The relevance of this strain and its nicotine catabolic pathway is twofold. First, the pathway is a source of functionalized pyridine intermediates such as 6-hydroxy-L-nicotine [11–13] and could be used as a renewable source of precursors to synthesize drugs and insecticides from nicotine or nicotine-containing waste [14]. Secondly and more relevant to this work due to being so well studied, the pAO1 megaplasmid and its nicotine degradation pathway provide a model for studying the molecular evolution of catabolic pathways and their spread by horizontal gene transfer via soil bacterial plasmids. The *nic*-genes cluster from pAO1 has a lower G + C content than the megaplasmid's average and integrases belonging to the tyrosine family of recombinases have been described at the cluster's 5′-end. These two findings suggest that the *nic*-genes DNA fragment could be a catabolic transposon [8] acquired by horizontal gene transfer [15].

Because pAO1 is a conjugative plasmid, reaching transfer frequencies of 10^–3^–10^–2^ per donor [7], it can also serve as a model for studying the molecular evolution of conjugative plasmids transmissible between related soil bacteria. Several attempts were made to classify the available plasmids associated with *Arthrobacter*-related species and to identify a backbone of core genes responsible for major plasmid functions such as replication, partition and conjugation [16–18]. Although such a core backbone remains elusive, several advancements have been made: four different clades of plasmids were described based on the sequence of the *ParA*-like protein [18], and a Type IV secretion system (T4SS) coupled with conserved DNA-repeats were identified within a subset of plasmids believed to have evolved from a common ancestor [19].

Nowadays, there are many sequencing projects looking at the microbial diversity of soil, especially in toxic or polluted samples [20]. *Arthrobacter*-related species have a high metabolic versatility and often constitute an important and even dominant culturable fraction of the microbial communities [21]. This has led to the accumulation of many *Arthrobacter*-related bacterial genomes. However, until 2016, within the *Arthrobacter* genus were grouped many species which were sequentially classified under 5 novel genera [4], including *Paenarthrobacter*. Therefore, classification of a given genome as belonging to the *Arthrobacter* or *Paenarthrobacter* species is dependent on when the data was deposited and if this taxonomic change was propagated in the databases containing rRNA sequences, leading to some confusion. Several comparative genomics studies dealing with *Arthrobacter* and *Paenarthrobacter* strains and focusing on identifying specific catabolic traits are available [22–29], but a genome-based phylogenetic study is missing so far.

In our previous work [30], we announced the availability of the complete genome sequence of *Paenarthrobacter nicotinovorans* ATCC 49919 and of a closely related lab strain named nic*-* lacking the pAO1 plasmid and hence unable to degrade nicotine. The current study is building upon this previously published work, further characterising the *P. nicotinovorans* ATCC 49919 complete genome and using it as reference to identify related strains containing the *nic*-genes cluster. Whole-genome-based phylogeny was performed using 64 *Paenarthrobacter*, *Arthrobacter*, *Rhodococcus* and *Nocardioides* genomes, which led to the identification of several mislabelled strains in the database. Moreover, we showed that five plasmids and two contigs belonging to *Arthrobacter* and *Paenarthrobacter* strains are virtually identical with pAO1, and we identified a highly syntenic *nic*-genes cluster on 5 plasmids, one contig and 3 different chromosomes. Further data is provided indicating that the *nic*-genes cluster consists of a putative catabolic transposon and two additional locally colinear blocks containing accessory genes. The data reported here can be used for transcriptomics and proteomics studies of nicotine degradation in *P. nicotinovorans* ATCC 49919 and opens the way for engineering the strain for improved conversion of nicotine and nicotine-containing waste into valuable chemicals*.* Moreover, the complete genome provides a much-needed reference sequence for nicotine degrading microorganisms (NDMs) that use the pyridine-pathway for the catabolic process and can be used for the assembly and comparative genome analysis of other strains belonging to the *Paenarthrobacter* genus.

## Results

### *Paenarthrobacter nicotinovorans* ATCC 49919 genome features

Hybrid assembly using Unicycler [31] yielded the complete genome sequence of *Paenarthrobacter nicotinovorans* ATCC 49919 with the general features listed in Supplementary Table 1. The genome consists of two replicons: a 4,316,184 bp circular chromosome with an overall G + C content of 63.2%, and a 165,141 bp circular megaplasmid with an overall G + C content of 59.7%. A total of 4,026 genes encoding 3,930 proteins, 23 pseudogenes, 54 tRNAs, 2 ncRNAs, 1 tmRNA, and 6 identical ribosomal operons were identified on the chromosome. In two instances, each three genes of two ribosomal operons are in proximity, being separated either by a single or three non-rRNA-related CDS, respectively. On the plasmid, a total of 145 genes encoding 138 proteins were identified.

eggNOG assigned 2421 Gene Ontology (GO) terms to 626 (15%) annotated genes, and 1,334 PFAM protein families to 3,338 (83%) of the total proteins. Two ABC-transporter related protein families make up 12% of the total number of genes with PFAMs assigned: 3-TM domain of the amino acid permease protein (IPR010065) and the ATP-binding domain of an ABC transporter-like protein (IPR003439). Clusters of Orthologous Groups (COGs) were assigned for 3,383 genes (93%), most being categorized as involved in amino acid transport (E), carbohydrate transport and metabolism (G) and transcription (Table 1).

**Table 1:** Distribution of COG categories in the complete genome of *P. nicotinovorans* ATCC 49919

COG Categories	Number of genes		
	Chromosome	pAO1	Total
Amino acid transport and metabolism (E)	**440**	**11**	**451**
Carbohydrate transport and metabolism (G)	**414**	**10**	**424**
Transcription (K)	**402**	**13**	**415**
Inorganic ion transport and metabolism (P)	**236**	**5**	**241**
Energy production and conversion (C)	**196**	**11**	**207**
Replication, recombination, and repair (L)	**176**	**11**	**187**
Translation, ribosomal structure, and biogenesis (J)	**180**	**0**	**180**
Signal transduction mechanisms (T)	**167**	**6**	**173**
Lipid transport and metabolism (I)	**171**	**1**	**172**
Cell wall/membrane/envelope biogenesis (M)	**161**	**2**	**163**
Coenzyme transport and metabolism (H)	**136**	**7**	**143**
Posttranslational modification, protein turnover, chaperones (O)	**109**	**3**	**112**
Secondary metabolites biosynthesis, transport, and catabolism (Q)	**98**	**3**	**101**
Nucleotide transport and metabolism (F)	**93**	**1**	**94**
Defense mechanisms (V)	**74**	**2**	**76**
Intracellular trafficking, secretion, and vesicular transport (U)	**46**	**4**	**50**
Cell cycle control, cell division, chromosome partitioning (D)	**45**	**2**	**47**
Cell motility (N)	**11**	**1**	**12**
Chromatin structure and dynamics (B)	**2**	**0**	**2**
Cytoskeleton (Z)	**1**	**0**	**1**
Function Unknown (S)	**693**	**21**	**714**

CRISPRCasFinder reported four genes encoding putative Cas3 proteins (Class 1, type III) and 3 CRISPR arrays. CARD returned hits to 16 antibiotic classes which were tested using the disk diffusion method. The strain was shown to be resistant to nalidixic acid (30 µg/disk), ceftriaxone (10 µg/disk), neomycin (30 IU/disk), spectinomycin (10 µg/disk), and susceptible to ampicillin (10 µg/disk), benzylpenicillin (10 IU/disk), cloxacillin (10 µg/disk), cefuroxime (30 µg/disk), chloramphenicol (30 µg/disk), erythromycin (15 µg/disk), gentamicin (10 µg/disk), kanamycin (70 µg/disk), vancomycin (30 µg/disk) and tetracycline (30 µg/disk).

### Other *Paenarthrobacter nicotinovorans* genomes

**Fig. 1:**
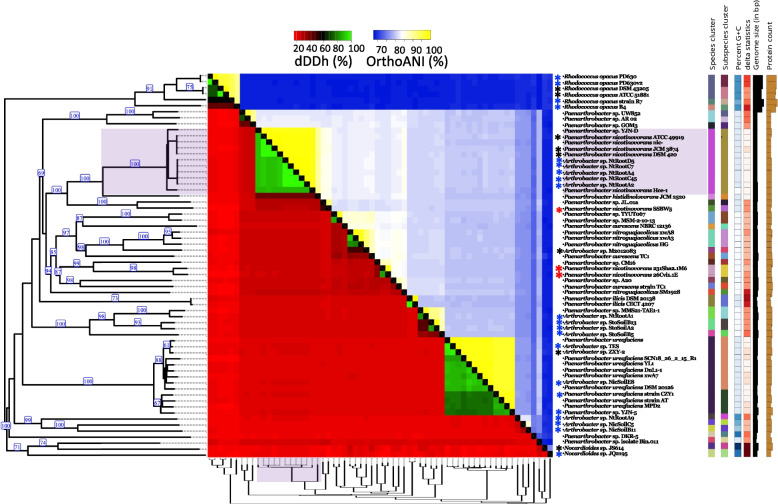
Phylogenetic tree and pairwise comparisons based on both dDDH (d_4_) and ANI values of 64 genomes of *Paenarthrobacter* strains and of other *Arthrobacter*, *Nocardioides,* and *Rhodococcus* strains which possess the *nic*-genes. The tree was inferred with FastME 2.1.6.1 [33] from GBDP distances calculated from genome sequences. The branch lengths were scaled in terms of GBDP distance formula d_5_. Values in blue represent GBDP pseudo-bootstrap support values > 60% from 100 replications, with an average branch support of 67.3%. The tree was rooted at the midpoint. The same tree clustering was used for both columns and rows in the heatmap. Cut-off values for species clustering was 70% for dDDH (d_4_) [34] and 96% for ANI. *Paenarthrobacter* strains mislabelled as *nicotinovorans* are marked with a red asterisk. Strains previously reported to harbour *nic*-genes are marked with a black asterisk. Strains first reported here to possess the *nic*-genes are marked with a blue asterisk

**Fig. 2:**
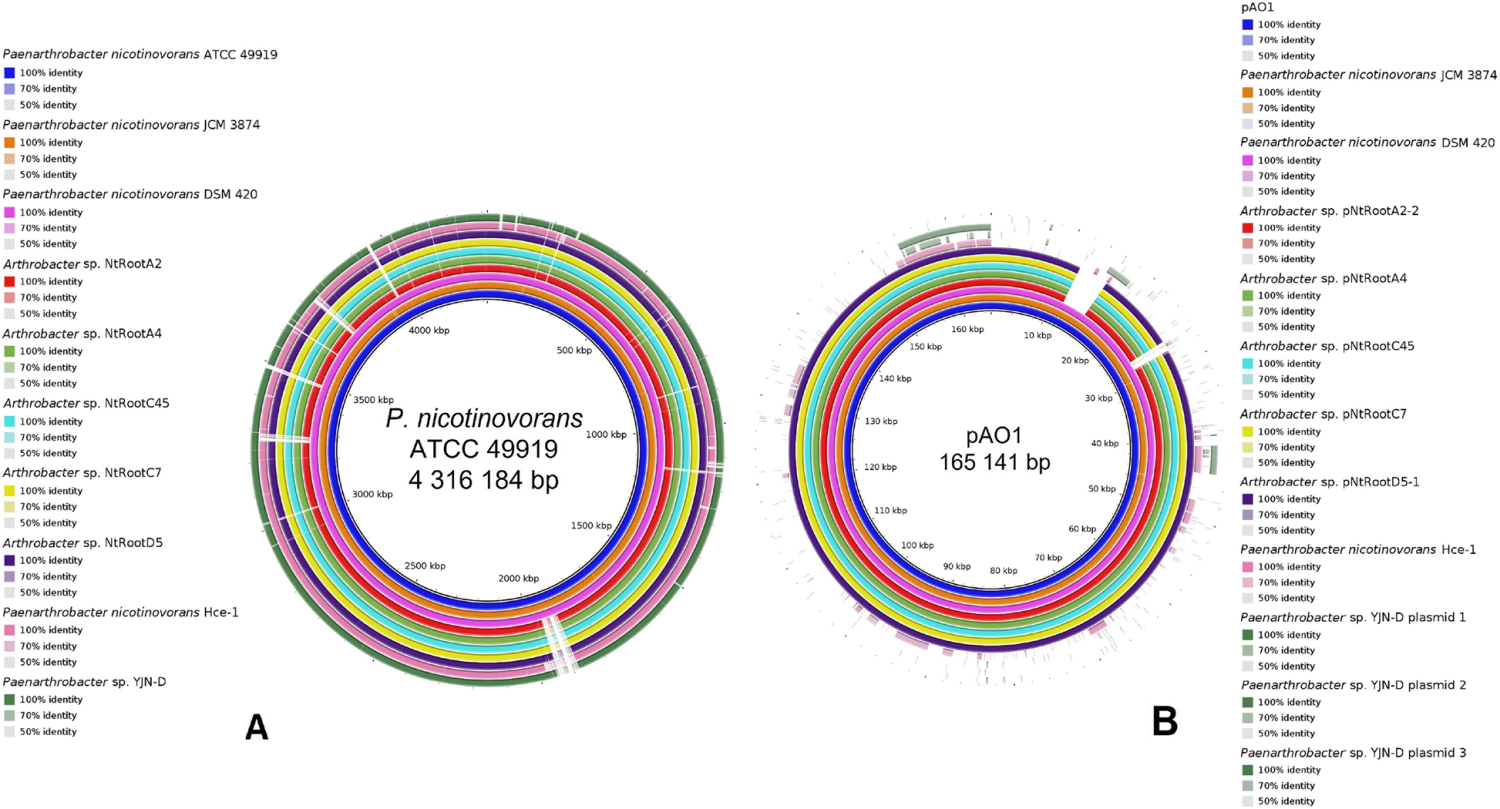
BRIG comparative genomic analysis showing the high similarity of the *P. nicotinovorans* ATCC 49919 genome with nine genomes of related *Arthrobacter* and *Paenarthrobacter* strains at the (**A**) chromosome and (**B**) plasmid level. The assembly IDs used for each strain are listed in Supplementary Table 2

The NCBI Genome database also lists eight more genomes from various *P. nicotinovorans* strains at contig or scaffold level (Supplementary table 2). As shown in Fig. 1, pairwise comparisons of the *Paenarthrobacter nicotinovorans* ATCC 49919 genome against these genomes indicated digital DNA-DNA hybridization (dDDH) values over the species threshold (calculated with formula d_4_) as well as average nucleotide identity (ANI) values over the proposed 96% species threshold [32] for 4 strains: *Paenarthrobacter nicotinovorans* nic*-*, *Paenarthrobacter nicotinovorans* JCM3874, *Paenarthrobacter nicotinovorans* DSM420 and *Paenarthrobacter nicotinovorans* Hce-1. The nic*-* strain is a cured derivative from ATCC 49919 lacking the pAO1 plasmid [30]. The strains JCM 3874, DSM 420 and ATCC 49919 are designations of the same strain and, as shown in Fig. 2, the corresponding deposited genomes contain a contig practically identical with pAO1 at nucleotide level. The draft genome of *Paenarthrobacter nicotinovorans* Hce-1 is highly similar at chromosome level with *Paenarthrobacter nicotinovorans* ATCC 49919, but not one of its 103 contigs is similar with the pAO1 plasmid or contains *nic*-genes. In line with these observations, GTDB-Tk assigned these strains as the same species based both on topological placement and ANI.

The strains below the threshold limit indicated by the TYGS phylogenomic analysis and GTDB-Tk as belonging to different species are *Paenarthrobacter nicotinovorans* SSBW5, *Paenarthrobacter nicotinovorans* 231Sha2.1M6 and *Paenarthrobacter nicotinovorans* strain 26Cvi1.1E (Fig. 1). The key characteristic of *Paenarthrobacter nicotinovorans*, i.e., the *nic-*genes, is also absent from the deposited genomes of these strains, indicating that these strains are mislabelled in the database. *Paenarthrobacter nicotinovorans* SSBW5 is more closely related to *Paenarthrobacter* sp. JL.01a (dDDH value 90%, ANI value 49%), while *Paenarthrobacter nicotinovorans* 231Sha2.1M6 and *Paenarthrobacter nicotinovorans* strain 26Cvi1.1E form a branch of their own. To remove further confusion, we propose that “*nicotinovorans*” should be dropped from the nomenclature of these three strains, and that they should be referred to as *Paenarthrobacter* sp. SSBW5, *Paenarthrobacter* sp. 231Sha2.1M6 and respectively, *Paenarthrobacter* sp. 26Cvi1.1E.

### Genomes of other nicotine-degrading microorganisms using the pyridine-pathway

The pyridine-pathway for nicotine degradation is not unique to *P. nicotinovorans* ATCC 49919, having been previously described in other NDMs [6] such as: *Arthrobacter* sp. ZXY-2 [8], *Arthrobacter* sp. M2012083 [25, 35], *Arthrobacter* sp. HF-2 [36], *Rhodococcus* sp. Y22 [37], *Rhodococcus opacus* DSMZ 43205 [38] and *Nocardioides* sp. JS614 [38]. Using the sequence of the 20 *nic*-genes experimentally related to nicotine degradation from pAO1 (bold entries in Supplementary Table 3), 21 more genomes (marked with a blue asterisk in Fig. 1 and listed in Table 2) were identified as containing at least 5 different *nic*-genes. As shown in Fig. 1, the phylogenomic analysis of the 21 strains indicated that 5 *Arthrobacter* sp. strains (NtRootD5, NtRootC7, NtRootA4, NtRootC45, NtRootA2) have both dDDH and ANI values over the species threshold when compared to *P. nicotinovorans* ATCC 49919. Moreover, all five *Arthrobacter* sp. strains contain a ~ 160.5 kb plasmid with high identity at the nucleotide level with pAO1 (Supplementary Table 4, pNtRootD5-1, pNtRootC7-1, pNtRootA4, pNtRootC45, and pNtRootA2-2, respectively), further suggesting that these strains actually belong to the same species as ATCC 49919 (Fig. 2, B). This was confirmed by the taxonomic assignment performed with GTDB-Tk, which also identified these strains as the same species.

**Table 2:** Genomes of strains harbouring at least 5 different *nic*-genes and their localization

**Organism Name**	**Strain**	**Assembly Accession**	***nic* genes found on**	
*Paenarthrobacter nicotinovorans*	ATCC 49919	GCA_021919345.1	pAO1	Identical *nic*-genes cluster
*Paenarthrobacter nicotinovorans*	JCM 3874	GCA_014648735.1	contig, BMRR01000008.1	
*Paenarthrobacter nicotinovorans*	DSM 420	GCA_017876445.1	contig, JAGINZ010000002.1	
*Arthrobacter* sp.	NtRootD5	GCA_019977175.1	pNtRootD5-1	
*Arthrobacter* sp.	NtRootC7	GCA_019977135.1	pNtRootC7-1	
*Arthrobacter* sp.	NtRootA4	GCA_019977095.1	pNtRootA4	
*Arthrobacter* sp.	NtRootC45	GCA_019977155.1	pNtRootC45	
*Arthrobacter* sp.	NtRootA2	GCA_019976995.1	pNtRootA2-2	
*Arthrobacter* sp.	NicSoilB11	GCA_019977375.1	pNicSoilB11-2	syntenic *nic*-genes cluster
*Arthrobacter* sp.	NicSoilE8	GCA_019977415.1	pNicSoilE8-2	
*Arthrobacter* sp.	TES	GCA_014863565.1	pTES1	
*Paenarthrobacter ureafaciens*	CZY1	GCA_016694995.1	pCZY	
*Arthrobacter* sp.	ZXY-2	GCA_001854365.1	pZXY21	
*Arthrobacter* sp.	M2012083	GCA_000281065.1	contig, NZ_AKKK01000058	
*Paenarthrobacter* sp.	YJN-5	GCA_015040095.1	chromosome	
*Arthrobacter* sp.	NtRootA9	GCA_019977115.1	chromosome	
*Arthrobacter* sp.	StoSoilB13	GCA_019977255.1	pStoSoilB13-1	*nic* genes spread within the replicon
*Arthrobacter* sp.	StoSoilA2	GCA_019977195.1	chromosome	
*Arthrobacter* sp.	NicSoilC5	GCA_019977395.1	chromosome	
*Arthrobacter* sp.	NtRootA1	GCA_019976975.1	chromosome	
*Arthrobacter* sp.	StoSoilB5	GCA_019977235.1	chromosome	
*Nocardioides* sp.	JQ2195	GCA_012272695.1	chromosome	
*Nocardioides* sp.	JS614	GCA_000015265.1	chromosome	
*Rhodococcus opacus*	R7	GCA_000736435.1	chromosome	
*Rhodococcus opacus*	B4	GCA_000010805.1	chromosome	
*Rhodococcus opacus*	PD630	GCA_000599545.1	chromosome	
*Rhodococcus opacus*	PD630	GCA_020542785.1	chromosome	
*Rhodococcus opacus*	DSM 43205	GCF_001646735.1	multiple contigs	
*Rhodococcus opacus*	ATCC 51881	GCF_012396235.1	multiple contigs	

### Highly syntenic *nic*-genes clusters identified in nine *Arthrobacter* and *Paenarthrobacter* genomes

Using pAO1 *nic*-genes as query, BLAST searches against the NCBI Nucleotide collection database identified a minimum of five *nic* genes on 12 plasmids, 14 chromosomes and 3 contigs from draft genomes belonging to strains from *Rhodococcus*, *Nocardioides*, *Arthrobacter,* and *Paenarthrobacter* genera. Plasmids pNtRootA2-2, pNtRootA4, pNtRootC7-1, pNtRootC45, pNtRootD5-1 as well as contig JAGINZ010000002.1 from the draft genome of *P. nicotinovorans* strain DSM 420 and contig BMRR01000008.1 from the draft genome of *P*. *nicotinovorans* strain JCM 3874 harbour a *nic*-genes cluster that is identical with the pAO1 one.

**Fig. 3:**
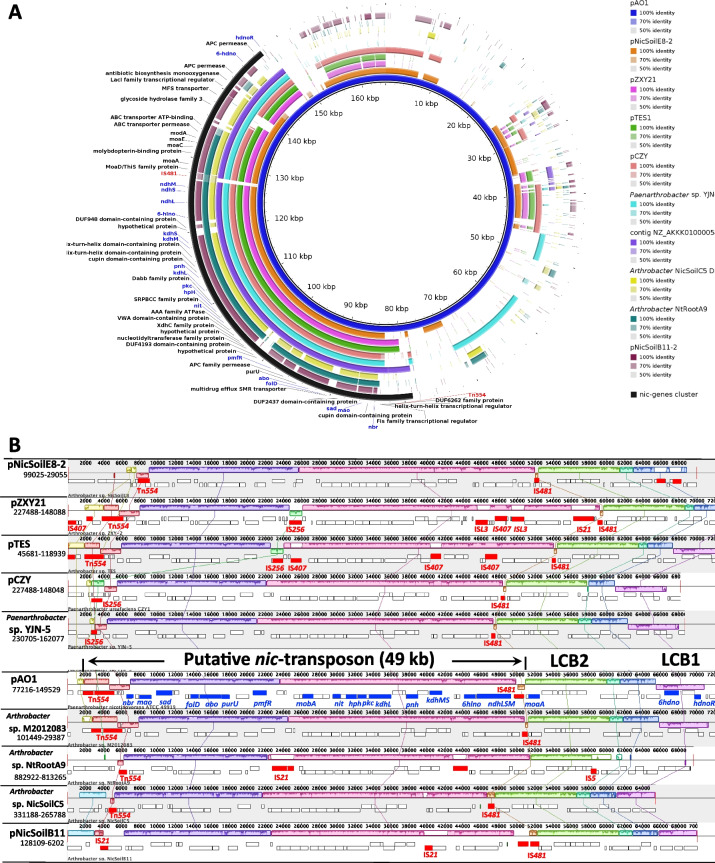
Nine highly syntenic *nic-*genes clusters identified in various *Arthrobacter* and *Paenarthrobacter* genomes. **A** BRIG comparative genomic analysis of the pAO1 megaplasmid against the strains harboring the *nic-*genes listed in the legend. External black circle – the localisation of the *nic*-genes cluster on pAO1; blue labels – pAO1 genes experimentally related to nicotine metabolism; red labels – genes related to recombination events. **B** Mauve alignments of the *nic*-genes clusters. Plasmids and genomes are listed on the left with the corresponding location of the *nic*-genes below. Coloured boxes – linear collinear blocks (LCB); white gaps – insertions and deletions; position atop or below the horizontal line represents the direction of LCB; red rectangles and text – recombination related ORFs; blue rectangles and text – pAO1 genes experimentally related to nicotine metabolism; *nbr* – nicotine blue oxidoreductase; *mao* – monoamine-oxidase; *sad* – succinic semi aldehyde dehydrogenase; *folD*—methylene-tetrahydrofolate dehydrogenase/ cyclohydrolase; *abo*—γ-N-methylaminobutyrate oxidase; *purU*—formyl-tetrahydrofolate deformylase; *pmfR*—transcriptional regulator; *mobA*—MobA, related to molybdopterin cytosine dinucleotide cofactor biosynthesis; *nit*—ω-amidase; *hph*—2,6-dihydroxypyridine-3-hydroxylase; *pkc*—putative polyketide cyclase; *kdhL*—ketone dehydrogenase, large subunit; *pnh*—2,6-dihydroxypseudooxynicotine hydrolase; *kdhMS*—ketone dehydrogenase, medium and subunits; *6hlno*—6-hydroxy-L-nicotine oxidase; *ndhLSM*—nicotine dehydrogenase, large, small and medium subunits; *moaA*—molybdopterin cofactor synthesis protein; *6hdno*—6-hydroxy-D-nicotine oxidase; *hdnoR*—transcriptional regulator; LCB2—locally colinear block containing genes associated with the synthesis of the molybdopterin cytosine dinucleotide cofactor; LCB1—locally colinear block containing genes for processing 6-hydroxy-D-nicotine. For an overview of the nicotine degradation pathway and role of each gene product, please see Supplementary Fig. 2

On the other hand, despite having lower overall nucleotide identity with pAO1, the following share a highly syntenic *nic*-cluster: plasmids pNicSoilB11-2, pNicSoilE8-2, pTES1, pCZY, and pZXY21, the chromosomes from *Arthrobacter* sp. NicSoilC5, *Arthrobacter* sp. NtRootA9 and *Paenarthrobacter* sp. YJN-5, as well as contig NZ_AKKK01000058 from the draft genome of *Arthrobacter* sp. M2012083 (Fig. 3, A). Comparative analysis performed with progressiveMauve indicated the presence of several locally collinear blocks (LCBs). Most of the *nic*-genes are placed within two LCBs and flanked by mobile genetic elements. At one end there are either transposases related to those of the *S. aureus* Tn*554* transposon [8], IS*21* or IS*256* family insertion sequences. At the other end the two LCBs are always flanked by the IS*481* family insertion sequence (Fig. 3, B).

## Discussion

The sequence of the two replicons comprising the complete genome of *P. nicotinovorans* ATCC 49919 offers a great opportunity to study the molecular evolution of the plasmid-encoded nicotine catabolic pathway and how it was spread to soil bacteria by horizontal gene transfer. The different G + C content of the pAO1 plasmid compared to the *P. nicotinovorans* ATCC 49919 chromosome suggests that the two replicons might have evolved independently [39] up to some point when the plasmid was acquired. Phylogenomic analysis of 64 related genomes identified 9 *Paenarthrobacter* and *Arthrobacter* genomes that have both dDDH and ANI values over the species threshold when compared to *P. nicotinovorans* ATCC 49919. Interestingly, two of these genomes, namely *Paenarthrobacter nicotinovorans* Hce-1 and *Paenarthrobacter* sp. YJN-D, do not contain any plasmids or contigs similar to pAO1. The phylogenetic tree depicted in Fig. 1 shows that *Paenarthrobacter* sp. YJN-D and *Paenarthrobacter nicotinovorans* Hce-1 share a common ancestor with all the strains harbouring the pAO1 megaplasmid. At some point, *Paenarthrobacter* sp. YJN-D or a closely related strain appears to have acquired the plasmid and the ability to metabolise nicotine, thus leading to ATCC 49919. Similar plasmid acquisition events detected by whole genome sequencing and by comparative genomics have been reported for the 167 kb plasmid pCFSAN061771_01 in *Escherichia coli* ST1485 [40], for two ∼40 kb plasmids in *Sulfurospirillum* sp. ACS_DCE_ and strain ACS_TCE_ [41], the ∼240 kb plasmid pS810b in *Pseudomonas aeruginosa* [42] and the much smaller 3.7 kb plasmid pPVER1 in *Providencia vermicola* [43].

The pAO1 plasmid sequence described here shows 99.99% identity with the previously available sequence of the pAO1 megaplasmid (GenBank entry AJ507836) but is 4 bp longer (165,141 bp vs 165,137 bp). The length difference provides a strong validation of our assembly by being in good agreement with the observation of the original pAO1 sequence submitters that the irresolvable compression they encountered during assembly could have increased the actual size of the plasmid by up to 5 bp [7]. Moreover, a closer look at the *nic*-genes cluster from the two pAO1 sequences indicated that all the genes that were experimentally related to nicotine metabolism were correctly annotated, with only minor differences for the position of the start or stop codons. The more distinct differences in annotations can be observed in the case of hypothetical and putative proteins, a situation that is due to improvements in gene detection algorithms (Supplementary Table 3).

**Fig. 4:**
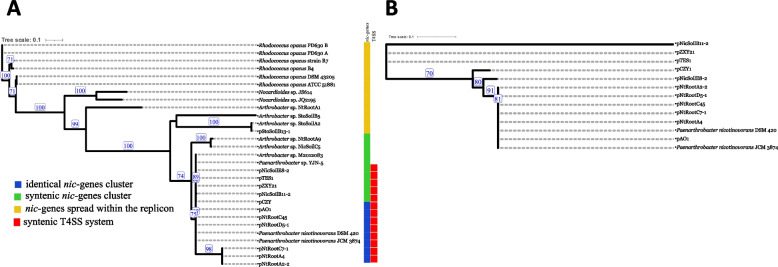
Maximum Likelihood phylogeny of the microbial pyridine pathway for nicotine degradation (**A**) and of the plasmids harbouring the *nic*-genes cluster **B**. The nicotine pathway phylogeny was reconstructed from the concatenated *purU, pnh, pmfR, ndhL, kdhL* gene sequences. The plasmids phylogeny was reconstructed from features postulated to be shared by *Paenarthrobacter* [19]: the syntenic T4SS system and DNA repeats located 5′ of 3 key ORFs—Duf4192, DprA and ParB. Numbers on branches indicate bootstrap support percentages and values > 70% are shown. The size bar corresponds to 0.1 nucleotide substitutions per site; the length of the dashed lines is not true to scale. Blue colour indicates identical *nic*-genes cluster; green indicates syntenic *nic*-genes cluster and orange indicates the presence of 5 *nic* genes, but the *nic*-cluster is not syntenic; red indicates plasmids that share a syntenic T4SS and DNA repeats located 5′ of 3 key ORFs—Duf4192, DprA and ParB

BLAST searches using the sequences of the *nic*-genes from pAO1 allowed the identification of 21 more genomes containing at least 5 different genes putatively related to nicotine metabolism. In an effort to identify a common ancestor for the *nic*-genes cluster from all these genomes, a phylogenetic tree was reconstructed from concatenated sequences of *purU*, *pnh*, *pmfR*, *ndhL*, *kdhL* (Fig. 4) and gene synteny was assessed (for an overview, see Supplementary Fig. 1). In 5 cases, the *nic*-genes are placed on plasmids that are almost identical with pAO1: pNtRootD5-1, pNtRootC7-1, pNtRootA4, pNtRootC45 and pNtRootA2-2 (dDDH values ≥ 99.7%, ANI values ≥ 99.9%, nucleotide identity levels in Supplementary Table 4). At about ~ 160.5 kb, all these plasmids are approximatively 4.6 kb smaller than pAO1, lacking two stretches of DNA that are outside of the *nic*-genes cluster: position 11,920 to position 15,907 encoding 5 hypothetical proteins, and position 26,501 to position 28,385 encoding a putative DprA DNA-processing protein reported as being involved in bacterial natural transformation [44].

A highly syntenic *nic*-genes cluster was identified in 9 *Arthrobacter* and *Paenarthrobacter* strains, all genes experimentally related to nicotine metabolism being located on three locally collinear blocks (LCBs) (Fig. 3, B). The two largest LCBs contain most of the *nic*-genes and are always flanked by integrases, further supporting the hypothesis that this is a catabolic transposon [8] which is spreading the *nic*-genes from/to chromosomes and plasmids in soil bacteria. The size of the transposon is 49 kb. There are other known similarly large transposons: the 56 kb Tn*4651* from *Pseudomonas putida* mt-2 responsible for toluene and xylene degradation, the 38 kb Tn*4655* from *P. putida* G7 responsible for naphthalene degradation and the 55 kb Tn*4371* from *Ralstonia oxalatica* A5 responsible for biphenyl/4-chlorobiphenyl degradation [45]. Mobilisation of the *nic*-transposon is controlled by 3 site-specific recombinases and transposases similar to Tn*544A*, Tn*554B* and Tn*554C* of the *S. aureus* transposon *544* and other predicted site-specific recombinases [8]. It is still unknown whether the mobilisation process of the *nic-*catabolic transposon is random or driven by other evolutionary forces. Nevertheless, it is generally accepted that the decision for transfer is under the control of the mobile genetic elements (MGEs) and that MGEs can sense when the cell is no longer a promising host for vertical transmission and leave the chromosome for horizontal transmission [46]. The invasion of this transposon by mobile elements that have been previously reported for plasmid pZXY21 [8] can also be seen for plasmids pTES and pNicSoilB11, and the chromosome of *Arthrobacter* sp. NtRootA9 (Fig. 3, B). Although the origin of this catabolic transposon remains elusive, the lower mean G + C content of these LCBs (58.03% ± 0.09) compared to the G + C content of the corresponding replicons/contigs (59.7% up to 67.2%) indicate that the *nic*-genes have been acquired by horizontal gene transfer by all these hosts. So far, we report the presence of this nic-transposon only in members of *Paenarthrobacter, Arthrobacter, Rhodococcus*, and *Nocardioides* genera. The high (51% – 75%) G + C content that members of Actinobacteria are known for might be a limiting factor for further horizontal travel of this transposon within the soil bacterial communities. The high GC content in a genome is usually corelated with specific promotors sequences as well as specific codon usage [47] which together might hinder the survival of the *nic* transposon and pAO1 megaplasmid in strains with lower GC content.

The third LCB (labelled LCB1 in Fig. 3, B) is placed outside of the putative *nic*-genes-containing transposon and contains only two genes known to be involved in nicotine degradation. One is *6hdno* which encodes a 6-hydroxy-D-nicotine oxidase involved in processing the D-nicotine that might form through the racemisation of the naturally occurring L-nicotine during the decay of the tobacco plant in the soil. The second gene encodes the transcriptional repressor of *6hdno* which discriminates poorly between 6-hydroxy-D-nicotine and 6-hydroxy-L-nicotine as inducers [48]. As L-nicotine is the main product in tobacco leaves, this LCB is not essential for nicotine catabolism, but might offer an advantage in ecological niches where both D- and L-nicotine are present.

Also, outside of the putative transposon and always associated with *nic*-genes in these genomes is LCB2, shown in Fig. 3, B. This block of DNA contains genes involved in the synthesis of the molybdopterin cytosine dinucleotide cofactor [49, 50] required for the assembly of two key enzymes in the pyridine-pathway for nicotine degradation: nicotine dehydrogenase and keto-dehydrogenase.

It was previously shown that a subset of 12 *Arthrobacter/Paenarthrobacter* plasmids share a common mechanism of plasmid partitioning and conjugation—a syntenic T4SS and DNA repeats located 5′ of 3 key ORFs—Duf4192, DprA and ParB [19]. The same markers are present on all plasmids harbouring the *nic*-genes cluster reported here and the tree depicted in Fig. 4 indicates their evolutionary relationships. The group of plasmids identical with pAO1 (pNtRootA2-2, pNtRootA4, pNtRootC7-1, pNtRootC45, pNtRootD5-1, contig JAGINZ010000002.1 from the draft genome of *P. nicotinovorans* strain DSM 420 and contig BMRR01000008.1 from the draft genome of *P*. *nicotinovorans* strain JCM 3874) have a common ancestor with pNicSoilE8-2.

## Conclusions

The complete genome of *Paenarthrobacter nicotinovorans* ATCC 49919 was established using a combination of short- and long-read sequencing followed by hybrid assembly, providing a much-needed reference genome for *P. nicotinovorans* strains as well as for other NDMs that use the pyridine pathway for nicotine degradation. Whole-genome-based phylogeny of 64 related genomes available in the NCBI Genome database identified five *Arthrobacter* strains that show both dDDH and ANI values over the species threshold when compared to *P. nicotinovorans* ATCC 49919, hence they are mislabelled in the database. Three genomes labelled as *Paenarthrobacter nicotinovorans* strains fall below the species threshold limits and, as indicated by both the TYGS phylogenomic analysis and GTDB-Tk assignment, belong to different bacterial species. Moreover, we have shown that the ATCC 49919 strain most probably evolved from *Paenarthrobacter* sp. YJN-D or a very closely related strain by acquiring the pAO1 megaplasmid.

Five plasmids and two contigs belonging to *Arthrobacter* and *Paenarthrobacter* strains were shown here to be virtually identical with the pAO1 plasmid of *Paenarthrobacter nicotinovorans* ATCC 49919. Moreover, a highly syntenic *nic*-genes cluster was identified on four plasmids, one contig and four chromosomes. The *nic*-genes cluster contains two major LCBs that apparently form a putative catabolic transposon. The other two LCBs harbour accessory genes that are either involved in the assembly of rare co-factor-containing proteins or related to a more efficient use of the nicotine stereoisomers available in the soil.

Although the origins of the *nic*-genes cluster and the putative transposon still elude us, the data presented here offers another snapshot into the evolution of plasmids harboured by *Arthrobacter* and *Paenarthrobacter* species and their role in the spread of metabolic traits by horizontal gene transfer among related soil bacteria.

## Material and methods

### Genome sequencing, assembly, annotation, and functional analysis

The complete *Paenarthrobacter nicotinovorans* ATCC 49919 genome was sequenced and assembled using a hybrid approach based on Illumina short-reads and ONT MinION long-reads. Complete protocols and technical details on genomic DNA extraction, sequencing and data processing are provided in our previously published genome announcement paper [30]. Functional analysis of the protein-coding genes was performed using the eggNOG [51, 52] mapper v2 online service available at: http://eggnog-mapper.embl.de/. Genome-wide comparison and visualization were performed with BLAST Ring Image Generator (BRIG) [53]. CRISPRCasFinder [54] available at https://crisprcas.i2bc.paris-saclay.fr/ was used to scan for CRISPR arrays and associated Cas proteins. The Comprehensive Antibiotic Resistance Database (CARD) [55] was used to identify putative antibiotic resistance genes. Resistance to the identified antibiotic classes was further confirmed in the lab using the disk diffusion method on Mueller Hinton agar plates as previously described [56].

### Nucleotide sequence accession numbers

The complete and functionally annotated genome is deposited in NCBI Genome with Accession numbers: CP089293 [57] for the chromosome and CP089294 [58] for the pAO1 megaplasmid. All SRA entries are available under the BioSample SAMN17383832 [59] in NCBI BioProject PRJNA693273 [60]. MIGS [61] mandatory information for the complete genome of *Paenarthrobacter nicotinovorans* ATCC 49919 is provided in Supplementary Table 1 and our genome announcement paper [30].

### Identification of other genomes of nicotine-degrading microorganisms using the pyridine-pathway

The sequences of the 22 *nic*-genes experimentally related to nicotine degradation in pAO1 were used to perform BLAST searches against the NCBI Nucleotide collection database (max E-value 0.0001, min identity 60%). The genomes containing hits for at least 5 different *nic*-genes were further analysed.

Comparative analysis of *nic*-genes cluster was performed with progressiveMauve [62] using the HOXD scoring matrix [63]. Mobilome analysis of the *nic*-genes clusters and surrounding areas was performed using MobileElementFinder v1.0.3 (Database version: v1.0.2) [64]. Concatenated sequence tree using the sequences of 5 *nic*-genes (*purU*, *pnh*, *pmfR*, *ndhL*, *kdhL;* Supplementary table 3 for details) were done in PhyML 3.0 [65] using the Jukes-Cantor substitution model.

#### Whole-genome-based phylogeny

A total of 64 genomes were downloaded from NCBI Genomes, representing all the available *Paenarthrobacter* genomes plus the *Arthrobacter*, *Nocardioides* and *Rhodococcus* strains previously or herein demonstrated as having *nic*-genes (details and accession numbers in Supplementary Table 2). Taxonomic classifications were performed based on the Genome Database Taxonomy (GTDB, r207) [66] using the GTDB-Tk (v.2.2.4) toolkit [67]. The genomes were uploaded to the Type (Strain) Genome Server (TYGS) [34] for whole genome-based pairwise comparisons and phylogenetic inference. All pairwise comparisons among the set of genomes were conducted using Genome Blast Distance Phylogeny (GBDP) and accurate intergenomic distances inferred under the algorithm 'trimming' and distance formula d_5_ [68]. Digital DNA-DNA hybridization (dDDH) values and confidence intervals were calculated using the recommended settings of the Genome-to-Genome Distance calculator (GGDC) 3.0 [68, 69]. The resulting intergenomic distances were used to infer a balanced minimum evolution tree with branch support via FASTME 2.1.6.1 including SPR postprocessing [33]. Species clustering was done using a 70% dDDH cut-off [34], while subspecies clustering was done using a 79% dDDH threshold as previously introduced [33]. Average nucleotide identity by orthology (ANI) values were calculated using the OrthoANI algorithm [70]. All phylogenetic trees were visualized using iTOL [71] while heatmaps were generated with Heatmapper [72]. Sequence manipulations and alignment visualisation were done in Geneious Prime 2022.2.2 (https://www.geneious.com/).

### Supplementary Information

**Additional file1: Supplementary Table 1.** MIGS mandatory information for the complete genome of Paenarthrobacter nicotinovorans ATCC 49919. **Supplementary Table 2.** Genomes of strains belonging to the Paenarthrobacter, Arthrobacter, Nocardioides and Rhodococcus genera evaluated in the study. The table lists all the Paenarthrobacter strains available in NCBI Genomes database as well as Arthrobacter, Nocardioides and Rhodococcus strains known to have the nic-genes. **Supplementary Table 3.** Annotations of the nic-genes cluster on the two pAO1 plasmid sequences. Bold indicates genes experimentally related to nicotine degradation. **Supplementary Table 4.** MEGA BLAST results of different plasmids and contigs against the pAO1 megaplasmid of P. nicotinovorans ATCC 49919.

**Additional file 2: Supplementary figure 1.** Overview of identity and gene synteny among the *nic* -genes containing strains of *Paenarthrobacter, Arthrobacter*, *Nocardioides* and *Rhodococcus* evaluated in this study. A. The *nic*-genes form a single locally collinear block (LCB, red) in strains harboring an identical *nic*-genes cluster. B. Most *nic*-genes are located in three LCBs (yellow, green and magenta) in strains harboring a syntenic nic-genes cluster. C. Five key nic-genes (*purU, pnh, pmfR, ndhL,* kdhL) could be identified in other strains, but many genes are missing and no syntenic nic-genes cluster can be described.

**Additional file 3: Supplementary figure 2.** Overview of the nicotine catabolic pathway of *Paenarthrobacter nicotinovorans*. CAPS AND BOLD letters indicate the intermediates: 6-HMM – 6-hydroxy-methylmyosmine; 6-HPON – 6-hydroxy-pseudooxynicotine; 2,6-HPON – 2,6-dihydroxypseudooxynicotine; 2,6-DHP – 6-dihydoxypyridine; MGABA - γ-N-methylaminobutyrate ; 2,3,6-THP - 2,3,6-trihydroxypyridine; NB -nicotine blue, 4,4‘,5,5‘-tetrahydroxy-3,3‘-diazadiphenoquinone-(2,2‘); CH2 TH4 - methylenetetrahydrofolate; GABA -γ-aminobutyric acid ; SSA - succinic semialdehyde, alpha-KGA - a-keto-glutaramate; alpha-KG - a-keto-glutarate ; CAPS indicate enzymes catalyzing the stepwise degradation of nicotine: NDH - nicotine dehydrogenase; 6HLNO - 6-hydroxy-L-nicotine oxidase; 6HDNO - 6-hydroxy-D-nicotine oxidase; KDH - ketone dehydrogenase; DHPONH - 2,6-dihydroxypseudooxynicotine hydrolase; DHPH – 2,6-dihydroxypyridine-3-hydroxylase NBOR – nicotine blue oxidoreductase; MABO - γ-N-methylaminobutyrate oxidase; FolD - methylene-tetrahydrofolate dehydrogenase/cyclohydrolase; PurU - formyl-tetrahydrofolate deformylase; MAO -monoamine-oxidase; AO – amine-oxidase; SsaDH - succinic semialdehyde dehydrogenase; PKC – putative polyketide cyclase; NIT - w-amidase.

## Data Availability

*Paenarthrobacter nicotinovorans* ATCC 49919 is available upon request or at ATCC. The sequencing data is available under NCBI BioProject accession number PRJNA693273. The genome assembled and annotated sequences have been deposited at NCBI GenBank under the following accession numbers: CP089293 for *P. nicotinovorans* ATCC 49919 chromosome and CP089294 for *P. nicotinovorans* ATCC 49919 megaplasmid pAO1. All data analysed during this study is included in this published article and its supplementary files.

## Abbreviations

ANI: Average nucleotide identity
COG: Clusters of Orthologous Group
dDDH: Digital DNA-DNA hybridization
GO: Gene Ontology
LCB: Locally collinear block
NB: Nicotine Blue
NDM: Nicotine degrading microorganism
ONT: Oxford Nanopore Technology
T4SS: Type IV secretion system
