# Supplementary material for: Characterisation of the Paenarthrobacter nicotinovorans ATCC 49919 genome and identification of several strains harbouring a highly syntenic nic-genes cluster
Source: BMC Genomics. 2023 Sep 11;24:536. doi: 10.1186/s12864-023-09644-3 (PMC10494377; doi:10.1186/s12864-023-09644-3)
Supplement: Supplementary file 2 — Additional file 2: Supplementary figure 1. Overview of identity and gene synteny among the nic -genes containing strains of Paenarthrobacter, Arthrobacter, Nocardioides and Rhodococcus evaluated in this study. A. The nic-genes form a single locally collinear block (LCB, red) in strains harboring an identical nic-genes cluster. B. Most nic-genes are located in three LCBs (yellow, green and magenta) in strains harboring a syntenic nic-genes cluster. C. Five key nic-genes (purU, pnh, pmfR, ndhL, kdhL) could be identified in other strains, but many genes are missing and no syntenic nic-genes cluster can be described. [file 12864_2023_9644_MOESM2_ESM.pdf]

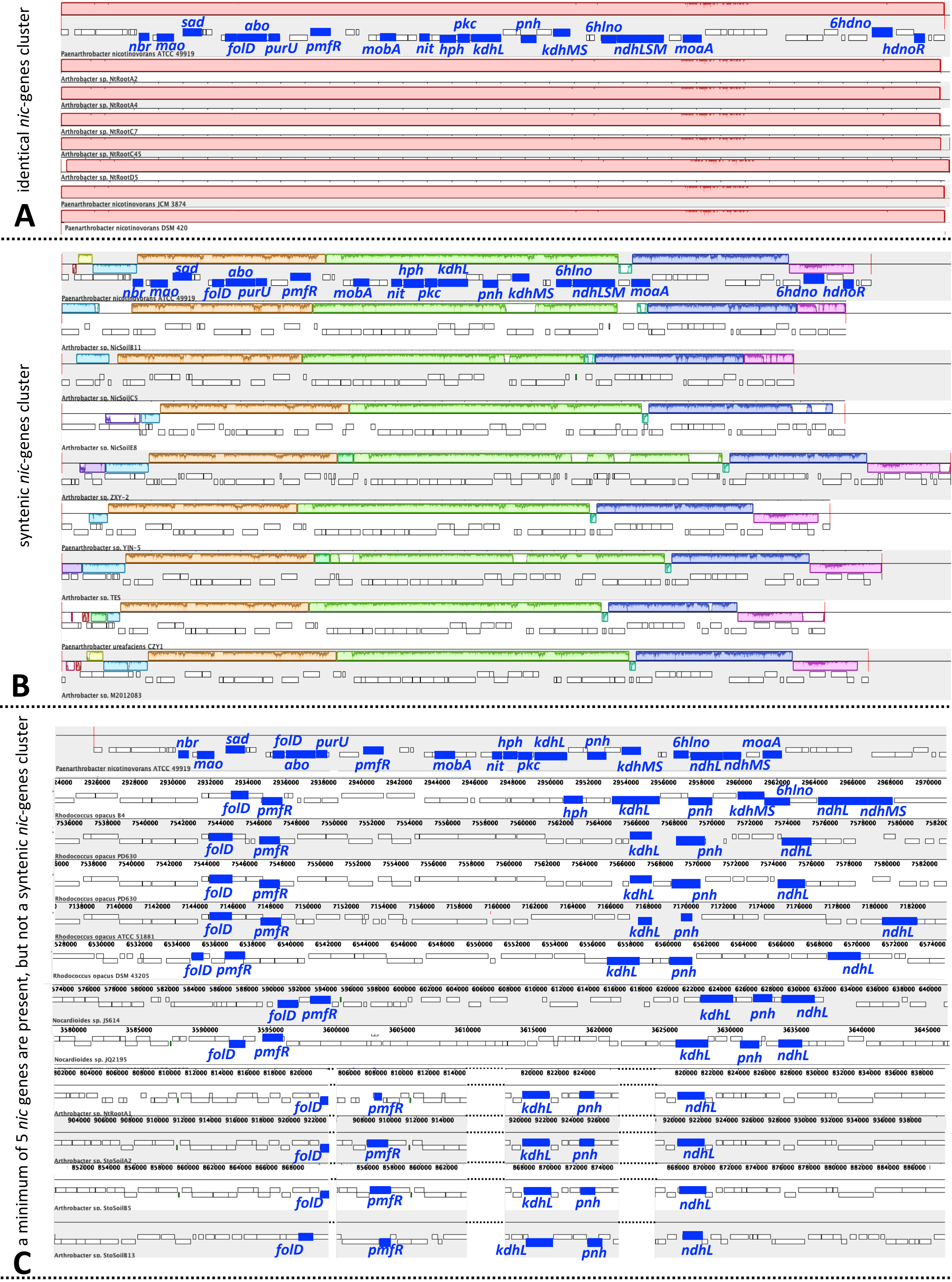

Supplementary figure 1. Overview of identity and gene synteny among the *nic* -genes containing strains of *Paenarthrobacter*, *Arthrobacter*, *Nocardioides* and *Rhodococcus* evaluated in this study. **A.** The *nic*-genes form a single locally collinear block (LCB, red) in strains harboring an identical *nic*-genes cluster. **B.** Most *nic*-genes are located in three LCBs (yellow, green and magenta) in strains harboring a syntenic *nic*-genes cluster. **C.** Five key nic-genes (*purU*, *pnh*, *pmfR*, *ndhL*, kdhL) could be identified in other strains, but many genes are missing and no syntenic nic-genes cluster can be described.
