# Supplementary material for: Characterisation of the Paenarthrobacter nicotinovorans ATCC 49919 genome and identification of several strains harbouring a highly syntenic nic-genes cluster
Source: BMC Genomics. 2023 Sep 11;24:536. doi: 10.1186/s12864-023-09644-3 (PMC10494377; doi:10.1186/s12864-023-09644-3)
Supplement: Supplementary file 3 — Additional file 3: Supplementary figure 2. Overview of the nicotine catabolic pathway of Paenarthrobacter nicotinovorans. CAPS AND BOLD letters indicate the intermediates: 6-HMM – 6-hydroxy-methylmyosmine; 6-HPON – 6-hydroxy-pseudooxynicotine; 2,6-HPON – 2,6-dihydroxypseudooxynicotine; 2,6-DHP – 6-dihydoxypyridine; MGABA - γ-N-methylaminobutyrate ; 2,3,6-THP - 2,3,6-trihydroxypyridine; NB -nicotine blue, 4,4‘,5,5‘-tetrahydroxy-3,3‘-diazadiphenoquinone-(2,2‘); CH2 TH4 - methylenetetrahydrofolate; GABA -γ-aminobutyric acid ; SSA - succinic semialdehyde, alpha-KGA - a-keto-glutaramate; alpha-KG - a-keto-glutarate ; CAPS indicate enzymes catalyzing the stepwise degradation of nicotine: NDH - nicotine dehydrogenase; 6HLNO - 6-hydroxy-L-nicotine oxidase; 6HDNO - 6-hydroxy-D-nicotine oxidase; KDH - ketone dehydrogenase; DHPONH - 2,6-dihydroxypseudooxynicotine hydrolase; DHPH – 2,6-dihydroxypyridine-3-hydroxylase NBOR – nicotine blue oxidoreductase; MABO - γ-N-methylaminobutyrate oxidase; FolD - methylene-tetrahydrofolate dehydrogenase/cyclohydrolase; PurU - formyl-tetrahydrofolate deformylase; MAO -monoamine-oxidase; AO – amine-oxidase; SsaDH - succinic semialdehyde dehydrogenase; PKC – putative polyketide cyclase; NIT - w-amidase. [file 12864_2023_9644_MOESM3_ESM.pdf]

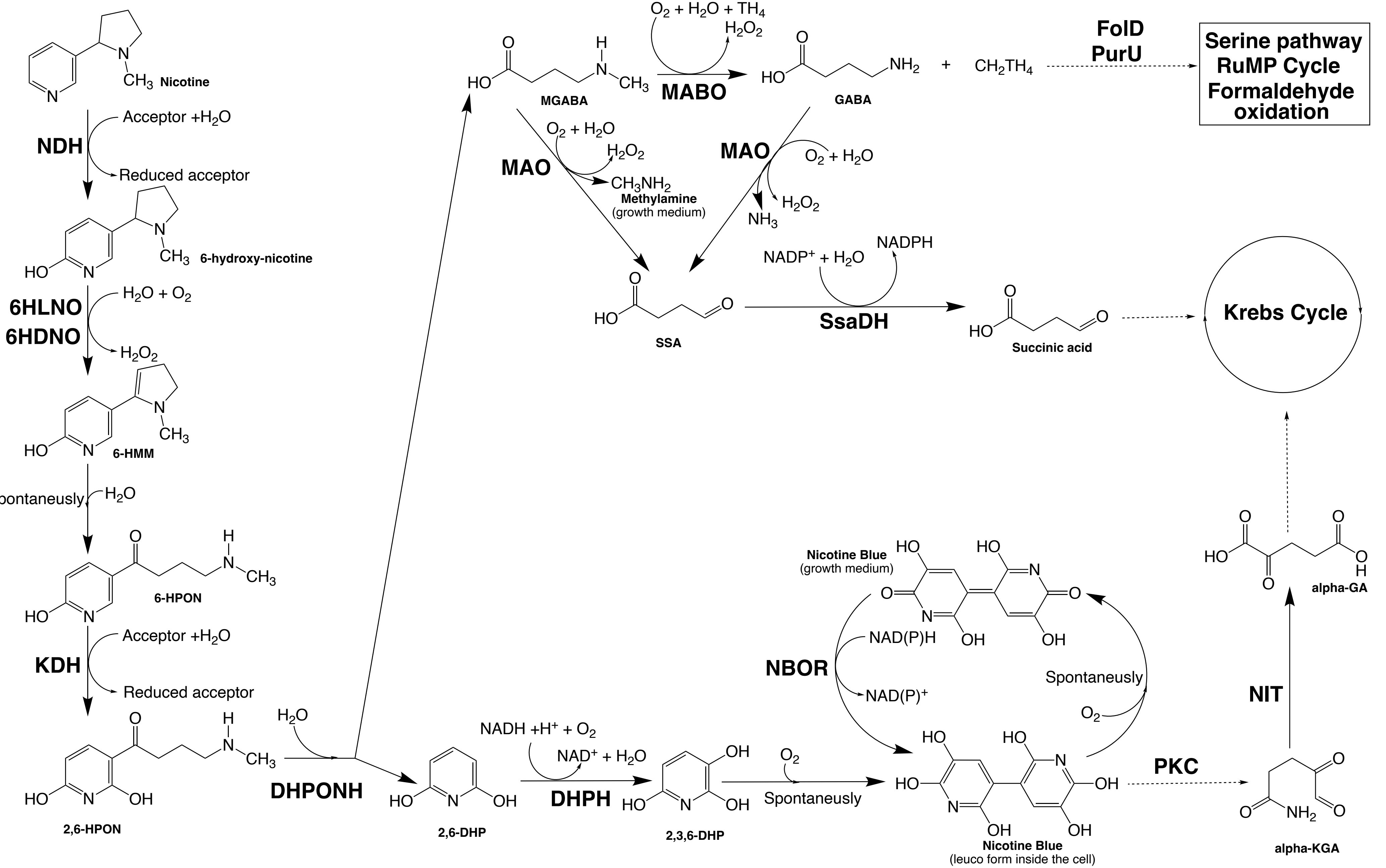

Supplementary figure 2. Overview of the nicotine catabolic pathway of *Paenarthrobacter nicotinovorans*. **CAPS AND BOLD** letters indicate the intermediates: 6-HMM – 6-hydroxy-methylmyosmine; 6-HPON – 6-hydroxy-pseudooxynicotine; 2,6-HPON – 2,6-dihydroxypseudooxynicotine; 2,6-DHP – 6-dihydoxypyridine; MGABA - γ-N-methylaminobutyrate ; 2,3,6-THP - 2,3,6-trihydroxypyridine; NB -nicotine blue, 4,4′,5,5′-tetrahydroxy-3,3′-diazadiphenoquinone-(2,2′); CH2 TH4 - methylenetetrahydrofolate; GABA - γ-aminobutyric acid ; SSA - succinic semialdehyde, alpha-KGA - α-keto-glutaramate; alpha-KG - α-keto-glutarate ;

CAPS indicate enzymes catalyzing the stepwise degradation of nicotine: NDH - nicotine dehydrogenase; 6HLNO - 6-hydroxy-L-nicotine oxidase; 6HDNO - 6-hydroxy-D-nicotine oxidase; KDH - ketone dehydrogenase; DHPONH - 2,6-dihydroxypseudooxynicotine hydrolase; DHPH – 2,6-dihydroxypyridine-3-hydroxylase NBOR – nicotine blue oxidoreductase; MABO - γ-N-methylaminobutyrate oxidase; FolD - methylene-tetrahydrofolate dehydrogenase/cyclohydrolase; PurU - formyl-tetrahydrofolate deformylase; MAO - monoamine-oxidase; AO – amine-oxidase; SsaDH - succinic semialdehyde dehydrogenase; PKC – putative polyketide cyclase; NIT - ω-amidase.
